# Case Report: Tuberculous meningitis presenting with multifocal cerebral infarction and hydrocephalus in a toddler

**DOI:** 10.3389/fped.2026.1866826

**Published:** 2026-07-02

**Authors:** Camila S. Contreras-Rojas, Agilda Dema, Henry David

**Affiliations:** 1 Department of Neurology, The University of Chicago Medicine, Chicago, IL, United States; 2 Department of Pediatric Neurology, The University of Chicago Medicine, Chicago, IL, United States

**Keywords:** acute ischemic stroke, hydrocephalus, inflammatory vasculopathy, *Mycobacterium tuberculosis*, pediatric neurology, tuberculous meningitis

## Abstract

**Background:**

Tuberculous meningitis is a severe pediatric emergency characterized by high rates of neurological disability, often due to hydrocephalus and inflammatory vasculopathy.

**Case description:**

We report a previously healthy 2-year-old boy who presented with a subacute febrile illness and, after interfacility transfer, sudden-onset focal neurological deficits. Initial diffusion-weighted magnetic resonance imaging on the day of admission revealed multiple predominantly unilateral punctate acute infarcts in the posterior limb of the internal capsule, thalamus, midbrain, and temporal lobe, despite a normal large-vessel CT angiogram and the absence of initial basilar enhancement or hydrocephalus. A cerebrospinal fluid analysis showed lymphocytic pleocytosis with profound hypoglycorrhachia, and the diagnosis was supported by a positive interferon-gamma release assay, cerebrospinal fluid mycobacterial nucleic acid amplification testing, and relevant epidemiological exposure, despite a normal chest radiograph; mycobacterial culture later confirmed a *Mycobacterium tuberculosis* complex. Follow-up imaging 4 days later documented rapid evolution to bilateral infarcts, diffuse basilar leptomeningeal enhancement, and obstructive hydrocephalus, demonstrating the temporal progression of disease. The patient was treated with a four-drug antituberculosis regimen and adjunctive corticosteroids, with ventriculoperitoneal shunt placement for hydrocephalus, following which he showed clinical improvement.

**Conclusion:**

This case highlights the phenomenon of clinical–radiological dissociation in early pediatric tuberculous meningitis, in which small-vessel vasculopathy and stroke may occur before classic meningeal enhancement is visible on imaging.

**Clinical significance:**

Maintaining a high index of clinical suspicion and initiating early empirical therapy based on cerebrospinal fluid profiles and epidemiological risk are crucial to preventing irreversible neurological outcomes.

## 1 Introduction

Tuberculous meningitis (TBM) in young children remains a critical medical emergency associated with a high risk of mortality and long-term neurodisability (1). The pathophysiology centers on the development of dense basal exudates that result in cranial neuropathies, obstructive hydrocephalus, and obliterative arteritis (2, 3). The patient case presented in this study is unique because it illustrates a significant initial “clinical–radiological dissociation,” in which focal neurological deficits and multifocal infarcts appeared before the classic findings of basilar enhancement or hydrocephalus were evident on neuroimaging (4).

## 2 Materials and methods

### 2.1 Diagnostic assessment

Initial evaluation included a non-contrast head computed tomography (CT) at the outside hospital that showed no acute intracranial abnormality. On the day of admission to our institution [hospital day (HD) 1], the acute onset of focal deficits prompted an ultrafast stroke magnetic resonance imaging (MRI), which demonstrated multiple punctate acute infarcts predominantly in the right hemisphere, involving the posterior limb of the internal capsule, thalamus and midbrain, anterior/medial temporal lobe, and lateral splenium of the corpus callosum on diffusion-weighted imaging (DWI) (Figure 1); immediate CT angiography of the head and neck excluded large-vessel occlusion. Given the fact that lesions tend to be bilateral on TBM, the radiological findings were unusual. Cerebrospinal fluid (CSF) evidenced WBC 70/μL with 87% lymphocytes, protein 184 mg/dL, and glucose 7 mg/dL and was found to have a positive test result for serum interferon-gamma release assay (IGRA) and a positive cerebrospinal fluid mycobacterial nucleic acid amplification test result (NAAT/PCR) for the *Mycobacterium tuberculosis* complex obtained from the diagnostic lumbar puncture. Human immunodeficiency virus (HIV) testing was negative. A chest X-ray did not evidence consolidations or opacities (Figure 2), and a transthoracic echocardiogram did not evidence endocarditis. A repeat contrast-enhanced MRI on hospital day 5 (4 days after the initial study) demonstrated new DWI lesions bilaterally (right pons, left thalamus, medial left temporal lobe) with progression of the right thalamic/splenial lesions (Figure 3A), new basilar leptomeningeal enhancement in the suprasellar and basal cisterns (Figure 3B), and early obstructive hydrocephalus (Figure 3C). A mycobacterial CSF culture, which was taken approximately two months later, confirmed *M. tuberculosis* complex infection.

**Figure 1:**
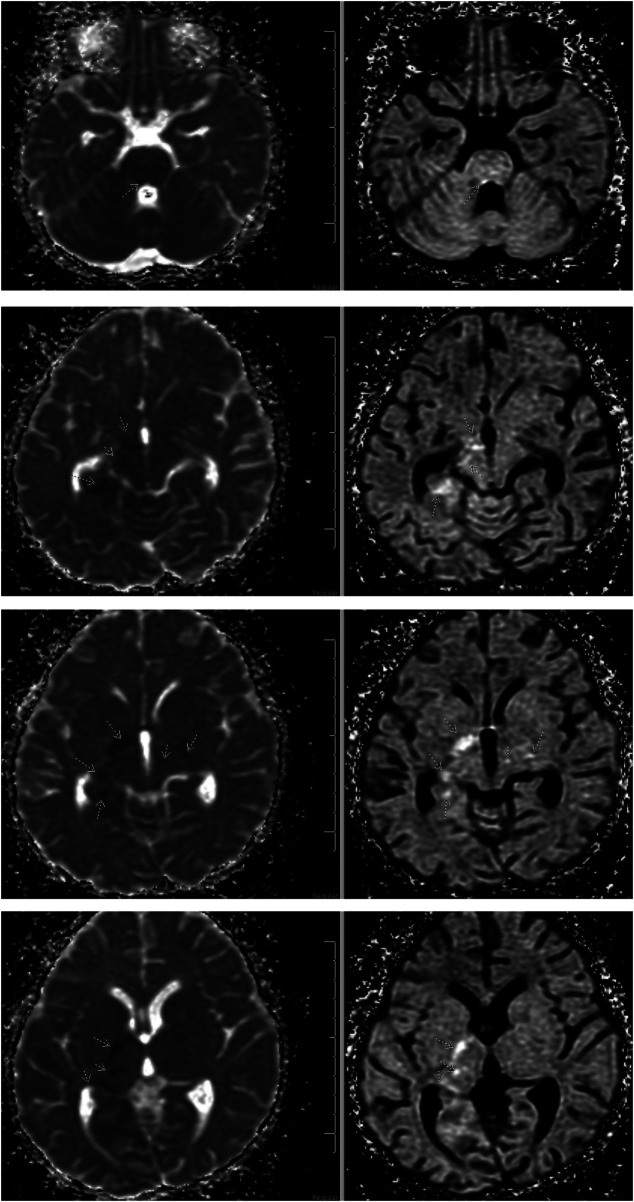
Initial MRI (hospital day 1): apparent diffusion coefficient (ADC) sequence/DWI showing punctate predominantly right-hemispheric infarcts (posterior limb of the internal capsule, thalamus/midbrain, temporal lobe), with normal ventricular size and no basilar enhancement. Retrospectively, scattered left deep punctate ischemic changes were also noted. The arrows indicate the acute infarcts.

**Figure 2:**
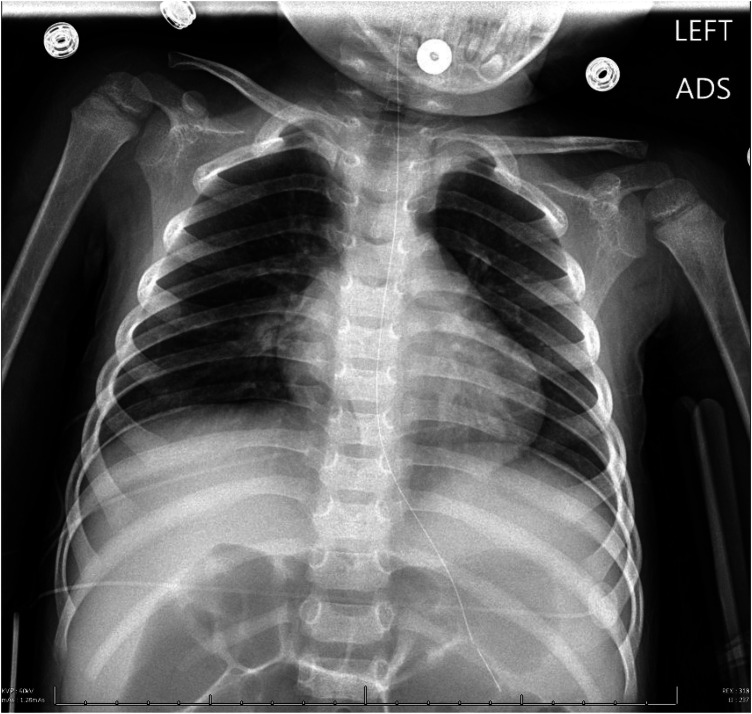
Admission chest X-ray (after the first MRI). There is no focus of consolidation, pleural effusion, or pneumothorax. The cardiac silhouette size is within normal limits. A normal cardiac apex and stomach are seen. Osseous structures are grossly unremarkable.

**Figure 3:**
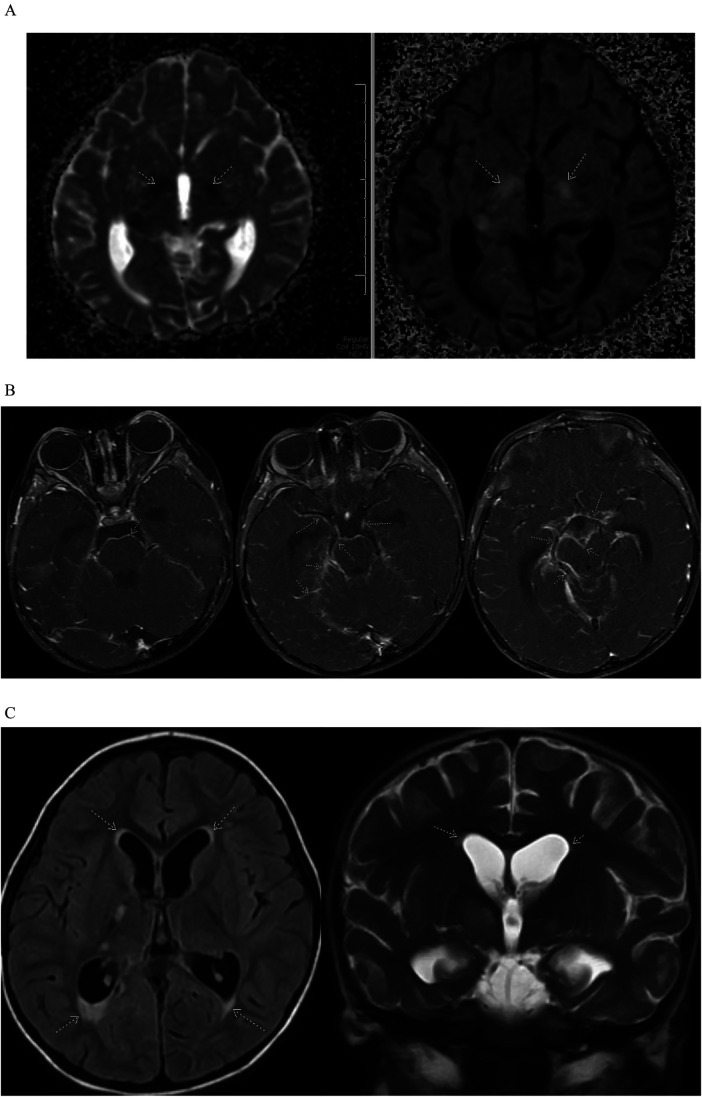
Brain MRI on hospital day 5 (4 days after the initial study). **(A)** Diffusion-weighted imaging shows new bilateral acute infarcts and increasing ventricular dilation, most marked at the occipital horns of the lateral ventricles. **(B)** Postcontrast imaging shows new basilar leptomeningeal enhancement in the suprasellar and basal cisterns. **(C)** Periventricular hyperintensity consistent with transependymal CSF flow [T2–fluid–attenuated inversion recovery MRI sequence (FLAIR) on the left and T2 on the right]. The arrows indicate the principal findings in each panel.

### 2.2 Ethics and informed consent

In accordance with Frontiers policies and CARE guidelines, written informed consent was obtained from the patient's legal guardians (parents) for the publication of this report and any accompanying images. All patient-identifying information has been removed to ensure anonymity.

## 3 Results

### 3.1 Presenting features and history

A previously healthy 2-year-old fully vaccinated US-born boy presented with persistent febrile illness (38.4–38.7 °C) associated with nausea, vomiting, lethargy, and decreased appetite approximately 9 days prior to admission to our institution. He was initially evaluated at urgent care and was discharged on oral Amoxicillin for possible acute otitis media. Despite the initiation of antibiotic therapy, fevers persisted, prompting return to the emergency department of an outside hospital the following day, where he was admitted for observation and rehydration. While being an inpatient, the fever curve intermittently improved, although it did not resolve, spiking hyperthermia up to 38.6–38.9 °C, and the patient continued to have altered mental status. The working differential at this stage included partially treated bacterial meningitis, herpes simplex and other viral meningoencephalitis, and a parainfectious or autoimmune encephalitis. Given the lack of improvement and progressive lethargy, empiric therapy for central nervous system infection was started with ceftriaxone (50 mg/kg IV), vancomycin (15 mg/kg IV q6h), and acyclovir (20 mg/kg IV q8h). A non-contrast head CT showed no acute intracranial pathology. Lumbar puncture under sedation demonstrated hypoglycorrhachia, hyperproteinorrhachia, and lymphocytic pleocytosis; the meningitis/encephalitis multiplex PCR panel, initial CSF bacterial cultures, and blood cultures were all negative. Two months after sampling, a mycobacterial NAAT/PCR performed on the same CSF specimen returned positive for a *M. tuberculosis* complex.

On the day of transfer to our institution following concerns about meningitis (hospital day 1), the boy developed sudden-onset acute bradycardia and lethargy, followed by sudden-onset right gaze preference, left nasolabial flattening, drifting of the left upper extremity, and decreased withdrawal to noxious stimuli on the left hemibody, activating a code stroke. An ultrafast stroke MRI evidenced the scattered punctate acute infarcts described above, and an immediate CT angiogram head and neck afterward ruled out large-vessel occlusion. He was admitted to the ICU for further monitoring.

The child was born in the US to parents from Guatemala; an uncle from abroad had recently stayed with the family. He was up to date on routine vaccines. His developmental history consisted of positive factors such as walking at 12 months, normal fine motor skills, speaking less than 10 words (naming objects but not speaking in full sentences), and being socially interactive. His mother had prenatal care in the US and was born full term without complications. He had a negative past medical history for central nervous system (CNS) trauma, motor vehicle collision, and unexplained loss of consciousness.

### 3.2 Clinical findings and timeline

On hospital day 2 (the day after admission), the patient was awake, arousable, and interactive although irritable, allowing an examination, with no verbalization. Left neglect and extinction were present. On a cranial nerve examination, he had left hemianopia with no blink to threat, anisocoria (right pupil 4 mm, left pupil 6 mm) round and reactive bilaterally, left ptosis, right gaze preference, and left nasolabial flattening with incomplete eye closure. A motor examination showed left hypotonia with deltoid abduction 1/5, biceps flexion 3/5, triceps extension 2/5, hand grasp 2/5, and 2/5 on hip flexion/extension and knee flexion/extension, with extensor plantar response on the right. There was no ataxia. There was left hyperreflexia on the patella and a left Babinski sign with inability to hold himself on standing position.

Over the following days, the boy’s mental status fluctuated with his fever curve: he appeared more lethargic, less interactive, and clinically ill during febrile episodes and more alert when afebrile. He developed an additional right cranial nerve III palsy and, later, bilateral extensor plantar responses. Serial imaging documented an evolving ventriculomegaly. The hydrocephalus was initially mild and of a communicating (malresorptive) pattern, and because the child remained neurologically stable on antituberculosis and corticosteroid therapy, cerebrospinal fluid diversion was deferred in favor of close clinical observation with serial MRI. When follow-up imaging showed persistent and then progressive ventricular dilation despite medical therapy, a navigation-guided right-sided ventriculoperitoneal shunt was placed on hospital day 15 (Figure 4). His neurological examination findings subsequently stabilized, and he was transferred to inpatient rehabilitation on hospital day 22. At a neurosurgery clinic visit approximately one month after admission, he was running and climbing around the examination room, moving all extremities spontaneously without clear facial paresis and with normal fine motor movements.

**Figure 4:**
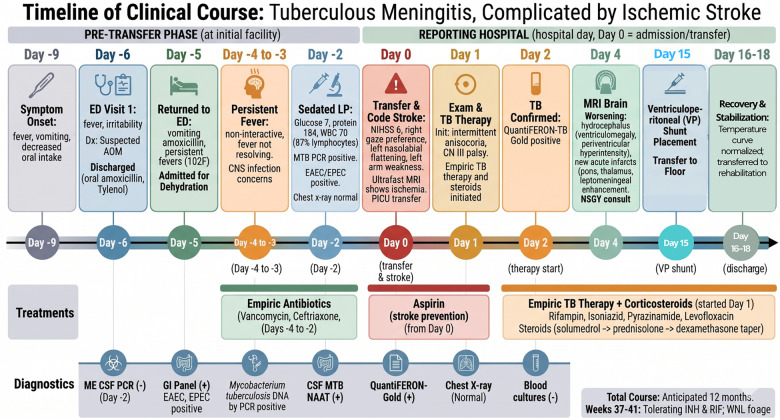
Timeline of clinical events, neuroimaging, and treatment by hospital day (HD). CT, computed tomography; CTA, CT angiography; MRI, magnetic resonance imaging; LP, lumbar puncture; CSF, cerebrospinal fluid; NAAT, nucleic acid amplification test; IGRA, interferon-gamma release assay; VPS, ventriculoperitoneal shunt; ASA, aspirin.

### 3.3 Therapeutic intervention

Empiric antituberculosis therapy was initiated on hospital day 2 with a four-drug regimen of rifampin (30 mg/kg/day), isoniazid (15 mg/kg/day), pyrazinamide (35 mg/kg/day), and levofloxacin (10 mg/kg twice daily), with coadministered pyridoxine; levofloxacin was substituted for ethambutol given its central nervous system penetration and the difficulty of monitoring visual acuity at this age. The planned total duration of therapy was 12 months (5–7). Adjunctive corticosteroids were administered, beginning with intravenous methylprednisolone (2 mg/kg/day), followed by oral prednisolone (2 mg/kg/day) on hospital days 2 through 5, and then dexamethasone (0.6 mg/kg/day) from hospital day 6 through discharge, with a subsequent gradual outpatient taper over 4–6 weeks. No worsening symptoms were described while decreasing steroids. Aspirin (40.5 mg daily) was started at the time of the stroke for secondary prevention, given the inflammatory vasculopathy characteristic of tuberculous meningitis, and was discontinued once ethology was established to be secondary to inflammatory vasculopathy. Adherence and safety were maintained through directly observed therapy in coordination with the Chicago Department of Public Health (using both home and video visits) and monthly laboratory monitoring, including liver function tests, to screen for drug-induced hepatotoxicity. Mycobacterial drug–susceptibility testing evidenced a pan-susceptible *M. tuberculosis* complex with plan for a total of 12 months of treatment. Household contact investigation identified a young cousin who tested positive for tuberculosis and was symptomatic and on a multidrug regimen consistent with active disease; the presumed index contact, an adult relative who had recently traveled from abroad, returned to his country of origin before testing could be completed. He completed the levofloxacin and pyrazinamide components at 2 months and continued isoniazid and rifampin, tolerating therapy without adverse effects.

## 4 Discussion

In toddlers with subacute fever, basilar meningitis, hydrocephalus, and perforator-territory infarcts, TBM should be presumed and treated immediately while diagnostic studies proceed. The combination of epidemiologic risk, characteristic CSF abnormalities, and MRI findings justifies prompt initiation of a four-drug antituberculosis regimen (rifampin, isoniazid, pyrazinamide, and a fourth agent—ethambutol or, as in this patient, levofloxacin) together with adjunctive corticosteroids and coordinated neurosurgical and rehabilitative care.

This patient case highlights that a normal MRA/CT angiography (CTA) does not exclude the small-vessel vasculopathy typical of TBM and that initial unilateral lesions on magnetic resonance angiography (MRI) should not rule out TBM. The strengths of this case study include the serial neuroimaging that captured the temporal evolution from isolated small-vessel involvement to classic basilar exudates from unilateral to bihemispheric ischemia (8, 9). A positive IGRA in a [Bacillus Calmette-Guérin (Tuberculosis vaccine) (BCG)-unvaccinated child provided strong support (10), and mycobacterial culture eventually confirmed *M. tuberculosis*. The patient demonstrated favorable clinical evolution, with resolution of the facial and left hemibody weakness after rehabilitation.

The imaging course in this child is best understood against the classic pediatric TBM pattern reported in prior series. Typically, neuroimaging demonstrates the triad of basal meningeal enhancement (most marked in the suprasellar and ambient cisterns), communicating hydrocephalus, and infarcts within the “TB zone”—the basal ganglia, internal capsule, and thalamus—arising from inflammatory involvement of the perforating arteries, with lesions that are frequently bilateral and often accompanied by tuberculomas (3, 4, 8). In contrast, our patient initially showed predominantly unilateral punctate infarcts in a perforator distribution in the absence of basilar enhancement, hydrocephalus, or large-vessel abnormality, with the classic basilar leptomeningeal enhancement, bilateral infarction, and obstructive hydrocephalus emerging only on repeat imaging 4 days later. This temporal sequence underscores the clinical–radiological dissociation that can characterize early disease and argues against using the absence of classic basal findings to exclude TBM (4, 9).

The biggest takeaway from this case study is that TBM must be presumed in toddlers with subacute fever and characteristic CSF findings, even if initial imaging lacks basilar enhancement or bilateral ischemic strokes (11–14).

## Data Availability

The original contributions presented in the study are included in the article/Supplementary Material, and further inquiries can be directed to the corresponding author.
